# Pre-departure and Post-elective Requirements for Global Health Electives: Survey of Canadian Royal College Emergency Medicine Programs

**DOI:** 10.7759/cureus.11680

**Published:** 2020-11-24

**Authors:** Jodie Pritchard, Susan A Bartels, Amanda Collier

**Affiliations:** 1 Emergency Medicine, McMaster University, Hamilton, CAN; 2 Emergency Medicine and Public Health Sciences, Queen's University, Kingston, CAN; 3 Emergency Medicine, Queen's University, Kingston, CAN

**Keywords:** emergency medicine, global health, residency electives, medical education, pre-departure training

## Abstract

Objectives: Global Health (GH) electives offer unique learning opportunities; however, risks to trainees and host populations should be minimized through pre-departure training and post-elective debriefing. In a 2016 study, only three Canadian residency programs mandated such training, although specific data on Emergency Medicine (EM) programs is lacking. This study aimed to identify GH elective requirements and perceived training gaps among EM programs.

Methods: We conducted two email surveys (one each for EM program directors [PDs] and residents) regarding training requirements and perceived gaps for GH electives. We also contacted university postgraduate medical education (PGME) and GH offices, via their online publicized emails, to assess university-wide requirements and resources.

Results: Nine PDs responded, with 78% reporting having residents participate in GH electives. Many PDs (67%) believed residents were moderately prepared for GH electives, while 33% felt they were unprepared to some degree. Forty seven out of an estimated 380 EM residents responded with 35% having completed a GH elective during residency. Of those, only one (6%) reported feeling very prepared, and 43% believed there was a need to improve trainings. Uncertainty around training requirements was reported, and residents identified challenges faced on electives, as well as priority topics for training. Responses from PGME and GH offices indicated that pre-departure training and post-elective debriefing were required or available at more universities than was indicated by the PD and resident respondents. However university requirements varied widely, with some exclusively requiring basic travel information and Health and Safety checklists or modules. The disparate responses indicate that residents and PDs may either be unaware of university requirements or not utilize available training resources for GH electives.

Conclusions: Although Canadian EM residents participate in GH electives, the majority of training programs do not require pre-departure training or post-elective debriefing. PDs and residents report varying levels of preparedness, and residents acknowledge a variety of challenges during GH electives. This information can be used to inform pre-departure training and post-elective debriefing and encourage EM residents to access available university-wide training.

## Introduction

The popularity of Global Health (GH) has surged over the last two decades. The availability of GH opportunities influences the ranking of medical schools and residency programs for many applicants, and approximately 25% of United States (US) undergraduate and postgraduate trainees complete a GH elective [1-2]. Similarly, 86% of US Emergency Medicine (EM) residents reported having incorporated the availability of GH electives into their residency program ranking decisions [3]. GH electives can increase trainee medical knowledge and skills, improve resourcefulness and cost consciousness, influence career paths, and provide an insight into ethical and societal issues [4-5]. A systematic review showed that GH electives increased the number of physicians seeking employment in lower income clinics as well as the number of trainees obtaining postgraduate education in Public Health [6]. However, these electives are not without risks, and several studies among organizations hosting students on GH electives document concern around patient safety, student preparation, cross-cultural skills, professionalism, understanding of local contexts, and resource drain [1,5].

Pre-departure training prior to GH electives is recommended by both the Canadian Federation of Medical Students (CFMS) and Resident Doctors of Canada (RDoC) [7-8]. The Liaison Committee on Medical Education has recently mandated pre-departure training as part of its accreditation standards [9]. Although pre-departure training was identified as essential in a 2016 Canadian Delphi study on elective requirements, no recommendations were made for post-elective debriefing by the committee. No standardized resident GH curricula exist, nor does the Accreditation Council for Graduate Medical Education (ACGME) have specific mandated criteria for residency programs offering GH electives, although some programs seek to have their electives approved to ensure credit toward residency requirements. Major groups including Consortium of Universities for Global Health (CUGH), Global Health Education Consortium (GHEC), and several major universities with GH programs have published recommendations for GH educational programs. As a common central theme across committees and institutions, robust pre-departure training and a focus on developing responsible, supervised electives seek to minimize negative impacts on host country resources, patients, and professional relationships, as well as on trainee health and well-being.

To the best of our knowledge, no published data exists on the pre-departure training and post-elective debriefing requirements of Canadian EM programs. To address this gap, we surveyed the Program Directors (PDs) and residents of all Canadian Royal College EM programs to identify the current GH elective training requirements, perceived gaps in training, and topics identified as important for inclusion in a pre-departure training course. Additionally, we contacted postgraduate medical education (PGME) and GH offices, via their listed email address on university websites, for each post-secondary institution with an EM residency program to document GH institutional training opportunities available for residents. This information can be used by EM programs to design or improve pre-departure training and post-elective debriefing for Canadian EM residents.

## Materials and methods

We conducted a cross-sectional survey of all 14 Canadian Royal College EM Residency programs to determine the current requirements for pre-departure training and post-elective debriefing for GH electives. GH electives were defined in the surveys as one that takes place in a country other than Canada, United States, United Kingdom, Western Europe, or Australia. Surveys were developed via consensus by the researchers using a standard Likert scale to assess level of preparedness. Items included as options for training topics were developed from the review of previously published literature and trainings. The 15-item PD survey and 22-item resident survey were written and delivered in English, and each was piloted by a number of emergency physicians prior to distribution.

The PD survey was emailed to the 14 PDs to determine the number of residents participating in GH electives. PDs were also asked which pre-departure training elements they considered important for their residents. The resident survey was sent to residents in Royal College EM programs, via email by each program’s chief resident. All current residents were invited to participate, in order to include the opinion and experiences of those who had and had not completed a GH elective, defined as in the PD survey. A link to the survey was also distributed to EM residents via the Canadian Association of Emergency Physicians (CAEP)’s resident newsletter. Resident surveys also asked participants about their awareness of required training for GH electives as well as which training topics should be included in pre-departure training and post-elective debriefs.

Surveys were distributed via email using Qualtrics survey software, with three reminder emails, over the period from December 2018 to February 2019. This study was approved by the Queen’s University Health Sciences and Affiliated Teaching Hospitals Research Ethics Board.

## Results

Between December 2018 and February 2019, nine of 14 EM PDs (64%) and 47 out of an estimated 380 residents from Royal College EM programs (12%) responded to the respective surveys. Results from each are presented below.

Program director surveys

Of PD respondents, 56% reported having one to two residents annually participate in GH electives, while 22% reported having either 0 or three to five residents participate yearly. Programs reported their residents participated in a range of electives, including trauma rotations (six programs), clinical work (five programs), course-based study (five programs), resident as educator/trainer (one program), and program development (one program).

Two programs reported pre-departure training, one delivered in an online format and one requiring in-person training (Table 1). Overall, 67% of PDs believed that their residents were moderately prepared, 11% slightly prepared, 11% moderately unprepared, and 11% very unprepared for GH work. Three (33%) respondents felt that improvements should be made to either pre-departure training or both pre-departure training and post-elective debriefing, while five (56%) were unsure if improvement was needed. A wide range of pre-departure training topics were felt to be important (Table 2).

**Table 1:** Program Director reported training requirements for Global Health electives

PD responses	Safety/risk assessment required?	Pre-departure training required?	Post-elective debriefing required?
Yes	3 (33%)	2 (22%)	0
No	2 (22%)	4 (44%)	7 (78%)
Don’t know	4 (44%)	3 (33%)	2 (22%)

**Table 2:** Ranking of importance of pre-departure Global Health training topics

Topics	Number of residents ranking as important to be included in pre-departure training	Number of PDs ranking as important to be included in pre-departure training
Safety and personnel security	34 (72%)	8 (89%)
Cultural sensitivity	33 (70%)	6 (67%)
Practice in low-resource settings	32 (68%)	5 (56%)
Scope of practice	29 (62%)	7 (78%)
Support during elective	29 (62%)	7 (78%)
Personal health	27 (57%)	7 (78%)
Ethics	27 (57%)	1 (11%)
Support after elective	18 (39%)	5 (56%)
Humanitarian systems	15 (32%)	2 (22%)
Other	1 (2%)	0

Resident surveys

Resident respondents included 24 males and 23 females (estimated response rate 12.4%). Most respondents (62%) were senior residents in PGY3-5. Sixteen residents had done a GH elective in medical school, and 18 reported GH work or volunteer experience prior to residency.

Approximately two thirds (66%) of residents had not completed a GH elective during their EM residency, 25.5% had completed one elective, and 8.5% had completed two to four electives. Residents reported a range of electives including clinical work (eight), trauma rotations (eight), education/training projects (four), program development (three), course-based study (one), and others (three). Reported elective locations included South Africa (seven) and one in each of Iraq, Malta, Laos, Nepal, Pakistan, and Namibia. Few residents reported required training, and many were unsure of the requirements when completing GH electives through their programs (Table 3).

**Table 3:** Resident reported training requirements for Global Health electives

Resident responses	Safety/risk assessment required?	Pre-departure training required?	Post-elective debriefing required?
Yes	11 (24%)	10 (23%)	6 (14%)
No	6 (13%)	11 (25%)	13 (31%)
Don’t know	28 (62%)	23 (52%)	23 (55%)

When completed, pre-departure training content varied across programs, and some topic areas were incompletely covered. Residents felt, however, that a wide range of pre-departure training areas were important prior to participating in a GH elective. Table 2 shows the topics perceived to be important for pre-departure training. Table 4 lists the topics perceived important to cover in a post-elective debrief.

**Table 4:** Resident ranking of importance of post-elective debriefing topics

Topics	Number of residents ranking as important to be included in post-elective debriefing
Difficult/stressful cases	35 (74%)
Challenges to working in lower resource setting	29 (62%)
Challenges with cultural differences	25 (53%)
Benefits of elective	24 (51%)
Skills/knowledge gained during elective	21 (45%)
Challenges related to lack of supervision	20 (43%)
Difficulty returning to Canadian practice	19 (40%)
Logistical challenges in arranging/completing elective	19 (40%)
Interpersonal challenges/conflict	18 (38%)
Unfamiliar medical topics	13 (28%)
Other	3

Of residents who completed a GH elective, only one (6%) reported feeling very prepared, 11 (69%) slightly to moderately prepared, and three (19%) reported they were either slightly unprepared or neither prepared/unprepared. No PD reported being made aware by a resident that they felt unprepared for their GH elective after their experience. However, residents did report several challenges related to their elective (Table 5).

**Table 5:** Resident reported challenges faced during Global Health electives

Topics	Number of residents reported experiencing during elective
Difficult coping with a different level of resources vs. home hospital	10 (63%)
Lack of supervision, making resident uncomfortable	5 (31%)
Difficult coping with death of patients	5 (31%)
Personal safety/security issue	3 (19%)
Expectation to do something outside scope of practice, resident uncomfortable	3 (19%)
Expectation to do something outside scope of practice, resident concerned for patient safety	2 (13%)
Lack of supervision, resident concerned for patient safety	2 (13%)
Personal medical issue	2 (13%)
Difficulty returning to Canadian practice	2 (13%)
Other challenging situations	2 (3%)

Eighteen residents (43%) reported there was a need to improve pre-departure training, post-elective debriefing, or both. Twenty one (50%) reported that they did not know if resources should be improved, three (7%) reported no need to improve resources, and five (11%) did not respond. Many topics prioritized by residents for post-elective debriefing related to managing challenges faced during GH electives. 

University PGME and Global Health Office information

PGME and GH Offices were contacted at universities which had an EM residency program. Emails were directed to the addresses publicly listed for each on their respective university web pages. Information obtained showed that six universities required pre-departure training, with five offering online modules. Six universities have post-elective debrief available in various forms, with three reporting mandatory in-person sessions. Information on the content of training was not available for all sites, with some focusing on Health and Safety modules and policies. For some schools, training is mandatory only at the undergraduate level but is offered as an option to residents.

## Discussion

Our study sought to understand perspectives regarding current requirements, perceived gaps in, and need for improved resources to support Canadian EM residents undertaking GH electives. Previous research has demonstrated unclear results as to the level of training offered to, and required of, residents prior to GH electives. A 2013 survey of US residency training programs found that 66% offered some pre-departure preparation, with 40% of EM programs offering this training [2,3]. Another 2013 study showed that only 15.4% of EM programs offered training for working internationally [10]. Data for Canadian residency programs is less clear, with one 2014- 2015 study showing that pre-departure training was mandatory in only three programs [11]. Our study demonstrated that few EM residency programs required pre-departure training, and none required post-elective debriefing. Although most programs have residents participating in GH electives, many PDs and residents were unsure of the training requirements. This suggests that even if pre-departure training and post-elective debriefing are available for residents, it may not be well communicated to postgraduate medical trainees or may not be considered essential for the elective.

Overall a low number of PD and resident respondents felt that they were not prepared for GH electives. Previous surveys of those hosting trainees on GH electives report that less than 60% were appropriately prepared [12], with some reporting that only 20% were well prepared [1], indicating that residents’ perceptions of their preparedness may not match host expectations. A survey of medical and nursing students in the United States showed that participation in pre-departure training was not associated with perceived preparedness unless it focused specifically on travel safety, personal health, cultural awareness, clinical and leadership skills [7]. The majority of EM residents in our survey felt key areas to be covered in pre-departure training included safety and security, cultural sensitivity, practice in low-resource settings, scope of practice, support during elective, personal health, and ethics. PDs had similar priorities, although fewer indicated that ethics was important in the training. International partners emphasize the need for trainees to demonstrate humility and to possess mature communication and collaboration skills; they also strongly emphasize the need for an understanding of culture, local healthcare practices, health workforce issues, and the link between human rights and health [12]. Resident responses emphasized the current demands of residency and hesitancy to add to their workload by completing extensive coursework in advance of GH electives. Responses stressed that information should be “simple and practical.” One resident commented on the need for any training to avoid “increased work for the sake of saying that our programs prepare us” and felt that focusing on practical information such as what personal protective equipment (PPE) to bring would be more useful. Another response highlighted their belief that the elective experience itself instilled cultural awareness and was less valuable as a pre-elective training topic. Literature around the concept of cultural safety emphasizes the practice of analyzing how power imbalances institutional relationships and how personal biases influence relationships with patients [13]. Understanding the concept of cultural safety and developing a reflective practice before embarking on GH electives could further assist residents in ensuring the delivery of appropriate care while on elective as well as enhance their experiences and learning from working with patients in different contexts. These resident comments suggest that some training modules may need to be modified to improve their relevance and acceptability to participants.

Three of nine PDs who responded felt revisions were needed to improve training for resident’s GH electives, while five were unsure if improvements were required. Forty-three percent of residents reported a need for improvement, and 50% were unsure. This may highlight the lack of familiarity with available training or uncertainty that training will actually improve preparedness for an elective. It is possible for a resident to be unaware of training opportunities, even if they had completed an elective. Similarly, PDs may delegate the process of complying with elective training requirements to residents to organize with PGME or GH offices. Many respondents in our surveys stated training was not required, while others were unsure of requirements. This contrasted with the responses received from PGME and GH offices, which indicated that pre-departure training and post-elective debriefing were available and required at many sites. The content and focus of trainings varied widely, with some focusing exclusively on Health and Safety modules and basic travel requirements. Pre-departure training opportunities appear to be more widely available than residents and PDs report; however, whether the training meets the needs of residents in preparing for GH electives has not been explored. It is possible that the administrative nature (emphasizing Health and Safety checklists or paperwork requirements) of some of these listed trainings led many residents and PDs to not consider them as part of a formal university PGME GH elective training program. Alternatively, residents and PDs may have been unaware of the available opportunities for training within the PGME and GH offices at their universities. Open source pre-departure training resources, such as the edX The Practitioners Guide to Global Health course, may be used by PGME departments to address the needs identified in our surveys to initiate or enhance their pre-departure training and post-elective debriefing programs.

Residents who had completed GH electives reported a variety of challenges, including difficulties managing with differing resources, scope of practice and supervision, death of patients, and personal safety issues while on GH electives. However, no PDs reported that residents had expressed feeling unprepared upon their return from an elective. Despite facing these challenges, very few electives required post-elective debriefings. Residents may be, in our opinion, reluctant to discuss issues and lack of preparedness with their PDs for fear of limiting future GH electives or may find other avenues to discuss their concerns. Post-elective debriefings and resources can help address some of the concerns raised by assisting with re-integration back into their home environment, solidifying learnings, and helping to identify and address mental health and ethical concerns in returning trainees. It would also serve to highlight issues that could be integrated into pre-departure training for future residents planning similar electives. The survey identified challenges that are likely to be encountered by residents on GH electives from any residency training program and on which pre-departure training should focus to prepare residents anticipate and address. Given the nature of EM electives, with high exposure to critically ill patients, high-risk procedures, and multi-casualty events, EM-specific pre-departure curricula could be developed and utilized by programs to supplement university-wide resident pre-departure training to help prepare EM trainees for the challenges encountered while participating in GH EM electives.

This study has several limitations, including the low response rate by residents as well as not obtaining responses from all EM PDs in Canada. Additionally, surveys were sent out only in English, while there are three universities with EM training programs in Quebec. The incomplete response rate may be missing programs with robust pre-departure training and post-elective debriefing requirements, although comparison with available information for postgraduate requirements at the various schools revealed similar results. In order to preserve respondent anonymity, we did not ask participants to identify their home university, and therefore we cannot determine if PD and resident responses match the postgraduate offices’ stated requirements around GH electives or whether they broadly represented EM programs across Canada. We also focused our surveys on programs with a Royal College EM program. Given the limited elective time available for Certificate of the College of Family Physicians - Emergency Medicine (CCFP-EM) programs in Canada, we expected the number of residents who participated in a GH elective during their year of training would be low. The survey is also subject to response bias, where residents interested in GH, or those who had completed GH electives, may have been more likely to respond than those with no interest or experience. However, 66% of resident respondents reported they had not completed a GH elective; thus, their perspective is represented in the results.

Our survey of EM residents participating in GH electives found that many were unaware of or did not have access to pre-departure training and post-elective debriefing opportunities. Participation in pre-departure training may help to address concerns around patient safety and resident preparation for electives, including cultural safety, professionalism, and understanding of local contexts. Post-elective debriefings may help to solidify knowledge and experience gained on electives, re-integrate into Canadian residency training, as well as serve as a source of support for difficult encounters or experiences. More broadly, we know that Canadian residents are participating in GH electives, and our survey of EM residents indicates that they may be doing so without the necessary training and support.

Future work should examine the broader state of Canadian residents’ training and preparation for GH electives. Research should not only identify the current opportunities and gaps in training but also examine best educational practices offered to residents globally. We identified that current university resources are not currently fully utilized; thus, further research would help to understand whether this is due to a lack of awareness of resources, belief that available resources do not meet perceived needs, or is seen as too onerous by residents. With competency-based medical education, there is an opportunity to develop not only pre-departure and post-elective debriefing training but to link the competencies gained by participating in a GH training course and elective to educational objectives.

## Conclusions

Although interest in GH electives is growing, development of GH electives and training in Canada lags behind that of the United States. A majority of EM residency programs surveyed do not require pre-departure training and post-elective debriefing for residents participating in GH electives. EM residents who had completed GH electives reported experiencing a variety of challenges, and improved pre-departure training and post-elective debriefing may help ensure that electives are conducted safely and benefit residents, patients, and host institutions. Some PDs and residents may be unaware of existing training opportunities through their universities or online. Existing training for GH electives should be identified at universities offering PGME education and enhanced where required to meet the needs of residents. Canadian EM residents should be encouraged and supported to obtain appropriate pre- and post-departure training for all GH electives in order to maximize benefits and minimize risks to trainees, patients, universities, and host institutions.

## Appendices

Supplement A: Program Director Survey

Q1 Does your program have:

☐      5 or more residents per PGY year 

☐      Less than 5 residents per PGY year

Q2  In the last 10 years, have 1 or more residents in your program participated in international electives in the following areas (check all that apply)?

*For the purposes of this survey an international elective is one that takes place in a country other than the Canada, USA, UK, Western Europe, Australia*  

☐      Trauma rotation 

☐      Clinical work 

☐      Course based study 

☐      Education/training projects (where resident is the educator/trainer) 

☐      Program development 

☐      Research

☐      Other ________________________________________________

Q3 In the last 5 years, how many residents per year, on average, participate in international electives?

☐      0

☐      1-2

☐      3-5

☐      6-10

☐      11-20

☐      > 20 

Q4 Prior to an emergency resident completing an international elective, is a safety/risk assessment required?

☐      Yes

☐      No

☐      I don't know 

Q5 If yes, a safety/risk assessment is required, who oversees the assessment?

☐      Residency program 

☐      Postgraduate office 

☐      Faculty of Medicine 

☐      Office of Global Health 

☐      I don't know 

☐      Other ________________________________________________

Q6 Prior to an emergency resident completing an international elective, is pre-departure training required?

☐      Yes, in person 

☐      Yes, online 

☐      No

☐      I don't know 

Q7 If yes, pre-departure training is required, who conducts the training?

☐      Online (list source if known) ________________________________________________

☐      Residency program 

☐      Postgraduate office 

☐      Faculty of medicine 

☐      Office of Global Health 

☐      I don't know 

☐      Other ________________________________________________

Q8 If yes, pre-departure training is required, which of the following topics are included (check all that apply)?

☐      Safety and personnel security 

☐      Ethics

☐      Cultural sensitivity 

☐      Humanitarian systems 

☐      Practice in low resource settings 

☐      Personal health 

☐      Scope of practice 

☐      Support during elective 

☐      Support after elective 

☐      I don't know 

☐      Other ________________________________________________

Q9 What topics do you feel are important to be covered in pre-departure training for international electives (check all that apply)?

☐      Safety & personnel security 

☐      Ethics

☐      Cultural sensitivity 

☐      Humanitarian systems 

☐      Practice in low resource settings 

☐      Personal health 

☐      Scope of practice 

☐      Support during elective 

☐      Support after elective 

☐      Other ________________________________________________

Q10 Following return from an international elective, is a post-elective debrief required?

☐      Yes

☐      No

☐      I don't know 

Q11 If yes, a post-elective debrief is required, who conducts the debrief?

☐      Program director 

☐      An ER attending (other than program director) 

☐      Residency program administrator 

☐      Post-graduate office 

☐      Faculty of medicine 

☐      Office of Global Health 

☐      It is done as part of an on-line program 

☐      Other ________________________________________________

☐      I don't know 

Q12 In your opinion, which of the following best describes the preparedness level of emergency medicine residents in your program when they undertake an international elective?

☐      Very prepared 

☐      Moderately prepared 

☐      Slightly prepared 

☐      Neither prepared or unprepared 

☐      Slightly unprepared 

☐      Moderately unprepared 

☐      Very unprepared 

Q13 On returning from an international elective, has a resident ever expressed to you that they felt unprepared for the experience?

☐      Yes

☐      No

Q14 Do you feel there is a need for improved pre-departure or post-elective resources for your residents completing international electives?

☐      Yes - improved pre-departure training 

☐      Yes - improved post-elective resources 

☐      Yes - both improved pre-departure training and post-elective resources 

☐      No

☐      I don't know 

Q15 Is there anything else you would like to share regarding pre-departure training or post-elective resources for emergency residents undertaking international electives?

Supplement B: Resident Survey

Q1 What is your current level of training?

☐      PGY1  

☐      PGY2  

☐      PGY3

☐      PGY4

☐      PGY5

Q2 Sex

☐      Male

☐      Female

☐      Other (specify if you wish)  ________________________________________________

☐      Prefer not to answer

Q3 Have you completed an international elective in medical school?

☐      Yes

☐      No

Q4 Not including medical school electives, have you had other international work or volunteer experience prior to residency?

☐      Yes

☐      No

Q5 Did you complete any type of pre-departure training prior to residency?

☐      Yes

☐      No

Q6 How many international electives have you completed during your emergency medicine residency?

☐       1

☐       2

☐       3

☐       4

☐       5

☐       6

☐      7 or greater   

Q7 If yes, you have completed international electives during your EM residency, please list the country or countries where they took place.

________________________________________________________________

Q8 During your emergency medicine residency, have you participated in an international elective in any of the following areas (check all that apply)?

☐      Trauma rotation 

☐      Clinical work

☐      Course based study

☐      Education/training projects (where resident is the educator/trainer) 

☐      Program development

☐      Research

☐      Other ________________________________________________

☐      I have not completed an international elective

Q9 Prior to completing an international elective, is a safety/risk assessment required?

☐      Yes

☐      No  

☐      I don't know  

Q10 If yes, a safety/risk assessment is required, who oversees the assessment?

☐      Residency program  

☐      Postgraduate office  

☐      Faculty of Medicine  

☐      Office of Global Health  

☐      I don't know  

☐      Other  ________________________________________________

Q11 Prior to completing an international elective, is pre-departure training required?

☐      Yes, in person 

☐      Yes, online 

☐      No

☐      I don't know 

Q12 If yes, pre-departure training is required, who conducts the training?

☐      Online (list source if known) _______________________________________________

☐      Residency program

☐      Postgraduate office  

☐      Faculty of medicine  

☐      Office of Global Health  

☐      I don't know 

☐      Other ________________________________________________

Q13 If you have completed pre-departure training (even if not required) during your emergency medicine residency, which of the following topics were included (check all that apply)?

☐      Safety and personnel security  

☐      Ethics  

☐      Cultural sensitivity   

☐      Humanitarian systems   

☐      Practice in low resource settings   

☐      Personal health   

☐      Scope of practice   

☐      Support during elective   

☐      Support after elective   

☐      I don't know   

☐      I have not completed pre-departure training  

☐      Other   ________________________________________________

Q14 Which topics do you feel are important to be covered in pre-departure training for international electives (check all that apply)?

☐      Safety & personnel security   

☐      Ethics   

☐      Cultural sensitivity   

☐      Humanitarian systems   

☐      Practice in low resource settings   

☐      Personal health   

☐      Scope of practice   

☐      Support during elective   

☐      Support after elective   

☐      Other   ________________________________________________

Q15 Following return from an international elective, is a post-elective debrief required?

☐      Yes   

☐      No   

☐      I don't know   

Q16 If yes, a post-elective debrief is required, who conducts the debrief?

☐      Program director   

☐      An ER attending (other than program director)  

☐      Residency program administrator   

☐      Post-graduate office 

☐      Faculty of medicine   

☐      Office of Global Health   

☐      It is done as part of an on-line program   

☐      Other   ________________________________________________

☐      I don't know   

Q17 If you have completed a post-elective debrief, what topics were included?

☐      Unfamiliar medical topics   

☐      Difficult/stressful cases   

☐      Challenges working in a lower resource setting   

☐      Challenges related to lack of supervision   

☐      Challenges with cultural differences   

☐      Interpersonal challenges or conflict   

☐      Difficulty returning to practice back in Canada   

☐      Logistical challenges in arranging or completing the elective   

☐      Benefits of elective   

☐      Skills or knowledge gained during elective   

☐      I have not completed a post-elective debrief  

☐      Other   ________________________________________________

Q18 Please select which topics you believe are important to be included in a post-elective debrief?

☐      Unfamiliar medical topics   

☐      Difficult/stressful cases   

☐      Challenges working in a lower resource setting   

☐      Challenges related to lack of supervision   

☐      Challenges with cultural differences   

☐      Interpersonal challenges or conflict   

☐      Difficulty returning to practice back in Canada   

☐      Logistical challenges in arranging or completing elective  

☐      Benefits of elective   

☐      Skills or knowledge gained during elective   

☐      Other   ________________________________________________

Q19 Which of the following best describes how prepared you felt while completing your international elective?

☐      Very prepared   

☐      Moderately prepared   

☐      Slightly prepared   

☐      Neither prepared or unprepared   

☐      Slightly unprepared   

☐      Moderately unprepared   

☐      Very unprepared   

Q20 During your elective, did you experience any of the following situations? (Select all that apply)

☐      Lack of supervision which made you uncomfortable  

☐      Lack of supervision which made you concerned for a patient's safety   

☐      An expectation to do something outside your scope of practice which made you uncomfortable   

☐      An expectation to do something outside your scope of practice which made you concerned for a patient's safety   

☐      Difficulty coping with a different level of resources compared to your home institution   

☐      Difficulty coping with the death of a patient or patients  

☐      A personal safety or security issue   

☐      A personal medical issue   

☐      Difficulty adjusting to practice back at your home institution   

☐      Other challenging situation not listed above:   ________________________________________________

Q21 Do you feel there is a need for improved pre-departure or post-elective resources for emergency medicine residents completing international electives?

☐      Yes - improved pre-departure training   

☐      Yes - improved post-elective resources   

☐      Yes - both improved pre-departure training and post-elective resources   

☐      No   

☐      I don't know   

Q22 Is there anything else you would like to share regarding pre-departure training or post-elective resources for emergency residents undertaking international electives or your international elective experiences?

___________________________________
